# Newly recognized turbidity current structure can explain prolonged flushing of submarine canyons

**DOI:** 10.1126/sciadv.1700200

**Published:** 2017-10-04

**Authors:** Maria Azpiroz-Zabala, Matthieu J. B. Cartigny, Peter J. Talling, Daniel R. Parsons, Esther J. Sumner, Michael A. Clare, Stephen M. Simmons, Cortis Cooper, Ed L. Pope

**Affiliations:** 1 National Oceanography Centre, University of Southampton Waterfront Campus, European Way, Southampton SO14 3ZH, UK.; 2 National Oceanography Centre Southampton, University of Southampton, Southampton SO14 3ZH, UK.; 3 Departments of Earth Sciences and Geography, Durham University, Durham DH1 3LE, UK.; 4 School of Environmental Sciences, University of Hull, Cottingham Road, Hull HU6 7RX, UK.; 5 Formerly at Chevron Energy Technology Company, 6001 Bollinger Canyon Road, San Ramon, CA 94583, USA.; 6 Department of Geography, Durham University, South Road, Durham DH1 3LE, UK.

## Abstract

Seabed-hugging flows called turbidity currents are the volumetrically most important process transporting sediment across our planet and form its largest sediment accumulations. We seek to understand the internal structure and behavior of turbidity currents by reanalyzing the most detailed direct measurements yet of velocities and densities within oceanic turbidity currents, obtained from weeklong flows in the Congo Canyon. We provide a new model for turbidity current structure that can explain why these are far more prolonged than all previously monitored oceanic turbidity currents, which lasted for only hours or minutes at other locations. The observed Congo Canyon flows consist of a short-lived zone of fast and dense fluid at their front, which outruns the slower moving body of the flow. We propose that the sustained duration of these turbidity currents results from flow stretching and that this stretching is characteristic of mud-rich turbidity current systems. The lack of stretching in previously monitored flows is attributed to coarser sediment that settles out from the body more rapidly. These prolonged seafloor flows rival the discharge of the Congo River and carry ~2% of the terrestrial organic carbon buried globally in the oceans each year through a single submarine canyon. Thus, this new structure explains sustained flushing of globally important amounts of sediment, organic carbon, nutrients, and fresh water into the deep ocean.

## Teaser

Runaway turbidity currents stretch into the deep ocean to form the largest sediment accumulations on Earth.

## INTRODUCTION

Turbidity currents are seabed-hugging flows driven downslope by the excess weight of suspended sediment. These flows form the largest sediment accumulations on Earth (*1*). Only terrestrial river systems carry similar volumes of sediment (*2*), although one turbidity current can sometimes transport more sediment than the annual global flux from all rivers combined (*3*, *4*). Understanding turbidity current structure and duration is important for mitigating the considerable hazard that they pose to expensive seafloor infrastructure, such as oil and gas pipelines (*5*, *6*), or the network of seafloor cables that now carries >95% of Internet and other global data traffic (*7*). Turbidity currents play a significant role in global carbon cycling and sequestration (*8*, *9*), supply important nutrients to deep-sea ecosystems (*10*), and ventilate the deep ocean with fresh water (*11*), whereas their deposits (called turbidites) host major petroleum reservoirs worldwide (*12*) and contain important archives of Earth’s geological past (*12*).

In contrast to many millions of direct observations of velocity and suspended sediment concentration in rivers (*4*), there are remarkably few direct measurements from turbidity currents (*2*). These submarine flows are notoriously difficult to monitor due to their relatively inaccessible location, often unpredictable occurrence, and ability to severely damage instruments placed in their path (*2*). This paucity of direct observations has meant that previous models for turbidity currents were based mainly on laboratory-scale experiments or inferred from deposited sediment layers. To make a step change in understanding of turbidity currents and of their wider impacts, there is a compelling need to measure key parameters within full-scale events. In particular, we need to understand the internal structure of these flows and how this structure then determines the flow evolution and duration.

**Fig. 1:**
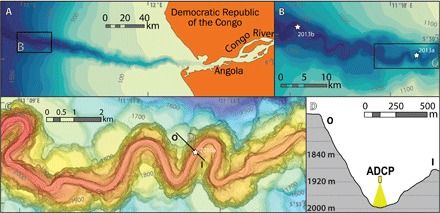
Mooring locations in the Congo Canyon. (**A**) Map of the Congo Canyon showing study area (rectangle), with bathymetric contours in meters. (**B**) Map showing the location of the two moorings deployed in 2013 (*6*, *57*). (**C**) Map showing location of 2010 mooring (*5*). (**D**) Cross-canyon profile showing ADCP suspended 85 m above the canyon floor. Location of cross section indicated in (C).

This study is based on the highest resolution measurements (made every 5 s) yet collected within an oceanic turbidity current (*5*, *6*). Initially shown by Cooper *et al*. (*5*, *6*), these measurements were collected in the Congo Canyon (*5*, *6*) at a water depth of 2 km using downward-looking acoustic Doppler current profilers (ADCPs) suspended 66 to 85 m above the seafloor (Fig. 1). Flows were measured at a single site from December 2009 to March 2010 (*5*), as well as at an additional site from January to March 2013 (Fig. 1, B and C) (*6*).

**Fig. 2:**
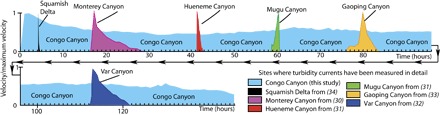
Turbidity currents that flush the Congo Canyon are far more prolonged than any previously monitored oceanic turbidity current. This figure compares the duration of the Congo Canyon flows studied here and oceanic turbidity currents that have been monitored previously using ADCPs in other shallower water locations (*30*–*34*).

A major surprise from both the 2009–2010 and 2013 measurements was that individual turbidity currents lasted for almost a week (Fig. 2 and Table 1), rather than hours or minutes as in all previous oceanic measurements from shallower water (Fig. 2) (*2*). Here, we present a new model of turbidity current structure that explains these prolonged flow durations. This model is based on a reanalysis of the ADCP data set collected by Cooper *et al*. (*5*, *6*), including the application of a novel acoustic inversion technique that provides us with a first insight into the distribution of sediment within individual flows (see Materials and Methods).

**Table 1:** Summary of flow properties of the 2010 deployment (numbering correspond to Fig. 3A).

**Flow**	**1**	**2**	**3**	**4**	**5**	**6**	**Mean**
Duration (days)	10.1	5.5	5.2	6.6	6.3	6.3	6.7
Maximum thickness (m)	53	57	48	69	77	68	62
Maximum ADCP velocity (m/s)	1.2	1.2	1	2.4	1.9	1.4	1.5
Average ADCP velocity (m/s)	0.4	0.4	0.3	0.7	0.5	0.4	0.5
Front propagation velocity (m/s)	0.8	0.8	0.7	1.6	1.5	1.0	1.1
Average height of maximum velocity above the bed (m)	6.8	6.9	5.8	14.2	11.8	10.0	9.3
Time of maximum velocity after arrival of the flow front (min)	25	34	100	8	25	25	36
Average sediment concentration (%_vol_)*	0.018	0.020	0.020	0.023	0.020	0.017	0.020
Peak sediment concentration (%_vol_)*	0.076	0.047	0.086	0.163	0.168	0.155	0.116
Maximum flow discharge (10^3^ m^3^/s)	4.6	4.9	2.7	14.9	16.3	10.4	9.0
Average flow discharge (10^3^ m^3^/s)	2.4	2.8	1.6	6.9	6.0	3.7	3.9
Maximum sediment discharge (10^3^ kg/s)*	3.1	2.7	2.0	13.2	9.0	6.1	6.0
Average sediment discharge (10^3^ kg/s)*	1.2	1.5	8.8	4.3	3.2	1.7	2.1
Water volume displaced (km^3^)	2.1	1.4	0.7	4.0	3.3	2.1	2.3
Sediment volume displaced (Mt)*	1.0	0.7	0.4	2.5	1.7	0.9	1.2
Organic carbon displaced (Mt)^†^	0.04	0.03	0.02	0.10	0.07	0.04	0.05

*Assuming a uniform grain size of 4.23 μm for inverting ADCP backscatter to sediment concentration (see Materials and Methods).

†Assuming an average carbon content of 3 to 5% weight, as measured within turbidity current deposits on the Congo Fan (see Materials and Methods) (*37*).

Our first aim is to document the internal structure of these turbidity currents, which is important because it determines how turbidity currents behave and evolve over time and space. Our second aim is to understand why these flows are so sustained. We show how their internal structure can explain their prolonged duration. Our third aim is to understand why the duration and character of these Congo Canyon turbidity currents differ from all previous measured oceanic turbidity currents and most laboratory experiments. We conclude by outlining the wider implications of this sustained flushing of submarine canyons for geohazards, organic carbon fluxes, and benthic ecosystems.

## RESULTS

**Fig. 3:**
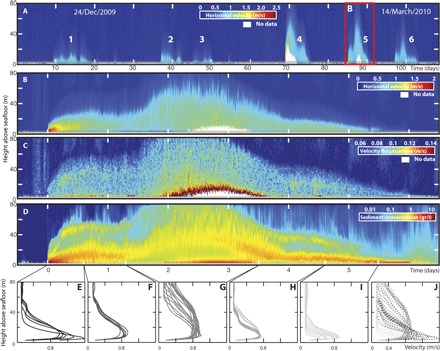
Turbidity current structure and duration from ADCP measurements at the 2009–2010 mooring site. (**A**) Full velocity time series for 3 months in 2009–2010 (*5*), showing that flows are active for ~33% of the time. Single flow in (B) to (D) is shown by the red box. (**B**) Horizontal velocity (30-min averages). (**C**) Intensity of large-scale velocity fluctuations, defined as the root mean square of differences between individual velocity measurements and 1-min averages. (**D**) Sediment concentrations inverted from 300- and 75-kHz ADCP acoustic backscatter (see Materials and Methods and figs. S3 to S10). This analysis assumes that the flow contains a single grain size, and variations in grain size may cause artefacts such as higher concentrations above lower concentrations. (**E** to **I**) Velocity profiles for different parts of the flow. (**J**) All velocity profiles combined in one plot.

Turbidity currents were active in the Congo Canyon for ~33% of the time during our 2009–2010 deployment period. Six prolonged flows dominate the 2009–2010 data set (Fig. 3A). The average flow lasted for 6.7 days, had a thickness of 62 m, and reached peak velocities of 1.5 m/s (Table 1). The highest velocities occurred at the front of the flow and are associated with maximum sediment concentrations (Fig. 3, B to D) of up to several tens of grams per liter (Table 1 and Fig. 3C). The velocity profiles over the remaining part of the flow showed considerable variation in their shape (Fig. 3, E to I), with peak velocities of around 0.8 to 1 m/s occurring between 6 and 14 m above the seafloor (Table 1). Average sediment concentrations are consistently about 0.02% by volume (Table 1). Peak sediment discharges of ~6000 kg/s (Table 1) exceed the sediment discharges of the Congo River and rival those of the Mississippi River (*4*). On average, a single turbidity current transports 2.3 km^3^ of water, ~1.1 to 3.8 Mt (million metric tons) of sediment, and ~0.03 to 0.19 Mt of organic carbon into the deep sea. Ranges in the amount of sediment and organic carbon (Table 1) reflect variations in the grain size and organic carbon content assumed (see Materials and Methods).

**Fig. 4:**
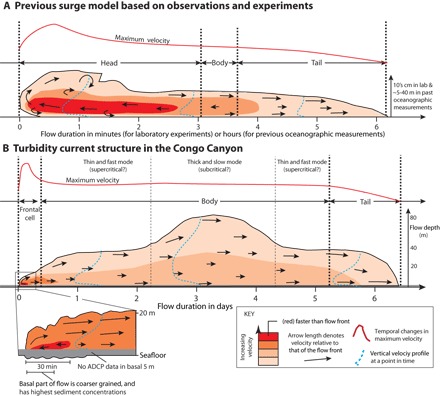
Turbidity current structure in laboratory experiments and in the Congo Canyon. Structure is shown by temporal changes in velocity measured at one spatial position, with red solid line denoting highest flow velocities. (**A**) Typical laboratory experiment with a finite-volume (surge-like) release (*13*–*16*) or previous oceanographic measurements (*30*–*34*); more sustained input would produce a better developed body. Arrows denote relative movement of sediment-laden fluid with respect to the flow front, with flow front velocity thus subtracted from measured velocities. The arrows show whether the body feeds the head. Temporal changes in maximum flow velocity are shown by red lines, and velocity profile shapes are shown by blue dotted lines. (**B**) Turbidity current structure in the Congo Canyon.

The internal structure of these turbidity currents in the Congo Canyon differs from that seen in previous experiments and measurements. On the basis of laboratory-scale experiments, previous models for sustained turbidity currents comprise an unsteady flow front (the head), followed by a steadier period of flow (the body) that finally wanes (the tail) (Fig. 4A) (*13*). The head is slower and thicker than the body because it has to displace surrounding water. Thus, the faster-moving body feeds the head with sediment-laden fluid (Fig. 4A) (*13*–*16*). The highest sediment concentrations occur in the head and lower part of the body; concentration, velocity, and flow thickness then decline within the tail of the flow (Fig. 4A) (*13*, *15*). The basic structure and hence behavior of turbidity currents in the Congo Canyon differ from these laboratory flows, and all previous measurements of surge-type oceanic turbidity currents (Fig. 2), in two important regards.

**Fig. 5:**
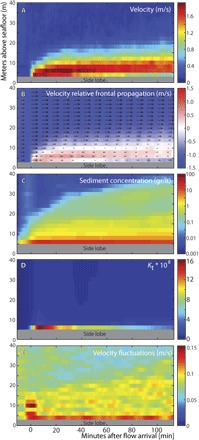
Structure of the frontal-cell during the first 120 min of the flow. (**A**) Horizontal flow velocity. Sidelobe interference area (see Materials and Methods) is covered by the gray box. (**B**) Velocity relative to the flow front propagation velocity (which is here fixed at 1.35 m/s). (**C**) Sediment concentration as derived from the acoustic inversion. (**D**) Variation of the calibration constant (*K*_t_) is used to indicate either grain size variation or higher sediment concentrations (see Materials and Methods). (**E**) Velocity fluctuations.

The first difference is that the Congo turbidity currents are composed of a short-lived zone (which we call the “frontal-cell”) of faster, dense, and coarse-grained flow (Figs. 3, 4B, and 5). Our ADCP backscatter inversion indicates that coarsest grains and highest concentrations are found within a few meters of the bed, close to the flow front (Fig. 5D). This frontal-cell runs away from the trailing body, unlike most laboratory experiments in which the body is faster than the head (*13*, *15*, *16*). The frontal-cell is thinner than the trailing body, and the velocity data show that the body is not feeding sediment-laden fluid into the frontal-cell (Fig. 3B). Instead, in our observations, the frontal-cell sheds sediment-laden fluid into the trailing body (Fig. 4B). This implies that the frontal-cell must continuously erode new sediment to replenish the sediment lost into the trailing body, making erosion of the canyon floor an important source of sediment. Calculations of bed shear stresses indicate that the frontal-cell can erode and incorporate sediment from the canyon floor, thus becoming self-sustaining (fig. S11) (*17*). Shear stresses beneath the trailing body are lower but still sufficient to suspend sand grains of up to ~200 μm (fig. S11).

We introduce the new terms, frontal-cell and trailing body, for the following reasons. Sequeiros *et al*. (*18*) used the term “head” for the self-sustaining frontal zone of a turbidity current. However, in the vast majority of previous studies, the head is not self-sustaining and has to be sustained by transfer of sediment-laden fluid from the body. This is not the case here. To prevent confusion, we use the term frontal-cell for a self-sustaining head. Our use of frontal-cell also emphasizes the observed circulation pattern (shown by arrows in Fig. 4B) in which fluid moves toward the flow front at the height of the velocity maximum, before being deflected upward and returning back toward the body in the uppermost and lowermost parts of the frontal-cell.

The second important difference is that the Congo Canyon flows are remarkably prolonged, with velocities of 0.8 to 1 m/s sustained for almost a week before dropping off in the tail of the flow (Figs. 2 and 3). This is in contrast to previous oceanic measurements in which an initial velocity peak is followed by a continuous decrease in velocity (Fig. 2). We propose a new model in which sustained flow is achieved because the frontal-cell outruns and feeds the trailing body, causing the flow to stretch (Fig. 4B). For example, if the flow front travels at 1.2 m/s, and the tail moves at 0.25 m/s, and both travel 170 km along the sinuous canyon, then the tail will arrive 6.2 days after the flow front at our measurement site. The amount of stretching depends on the relative values of the flow front and tail velocities, and reasonable values of these two velocities suggest stretching on the order of days (fig. S14). Flow stretching has the potential to generate ever more continuous turbidity currents further from the source, because slower moving events can be overtaken by faster moving ones, thus merging into a single longer event. However, previous studies of the deepest (>3.4 km) parts of the Congo Canyon have observed powerful but less frequent flows (*19*), suggesting that some flows die out in the lower canyon.

**Fig. 6:**
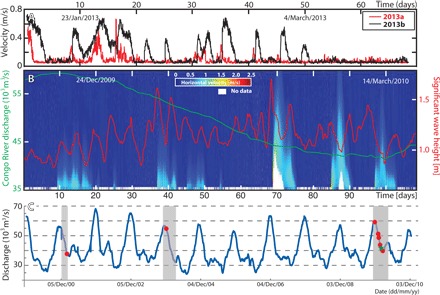
Timing and triggers of turbidity currents. (**A**) Flow velocities measured at 18 m above the seafloor by ADCPs at two mooring sites in 2013. Site 2013a is located 22-km up-canyon from site 2013b (Fig. 1B) (*6*). (**B**) Turbidity currents of the 2010 deployment plotted against two potential triggers: significant wave height in red and Congo River discharge at the Kinshasa gauging station in green. (**C**) Plot of changes in Congo River discharge (blue line), periods when measurement instruments were present in the canyon (gray bars) (*5*, *19*, *58*), and timing of turbidity currents (red dots). The green star is turbidity current shown in Fig. 3.

This stretching model has then been tested using a second ADCP data set collected from January to March 2013 (*6*). The duration of these 2013 flows was compared at two sites along the Congo Canyon that are 22 km apart (Fig. 1B) (*6*). Flow durations at the two moorings are shown in Fig. 6A and table S1. ADCP measurements in the lowermost 18 m of these flows are lacking due to reflections from the canyon wall. This is because the January-March (2013) moorings were located close to the canyon wall, although still within the flat canyon floor (see fig. S1). Nonetheless, at 18 m above the seafloor, the duration of these 2013 flows increased substantially in a down-canyon direction by an average of 0.74 ± 0.6 days (Fig. 6A and table S1). We note that the 2013 flows tend to be thicker at the downstream mooring, and this may also partly explain their longer duration at the downstream site (table S1). However, this significant increase in measured flow durations between the two sites in 2013 generally supports the hypothesis that these flows stretched as they moved down-canyon.

## DISCUSSION

We first discuss whether the prolonged turbidity currents of the Congo Canyon result from sustained sediment sources or from internal flow stretching. We then seek to understand why these turbidity currents in the Congo Canyon differ significantly from most (but not all) laboratory experiments, and all previous measurements from full-scale oceanic flows. Finally, we outline the wider implications of these sustained turbidity currents for carbon burial and geohazards.

### How were these flows triggered?

It is important to determine whether the prolonged duration of these flows results from a sustained initial source of sediment, as opposed to stretching of the flow as it moves down-canyon. Several potential triggers can be eliminated for these Congo Canyon flows (also see Materials and Methods). The flows were not triggered by earthquakes, because no significant earthquakes [moment magnitude (*M*_w_) > 2.5] occurred within 300 km of the canyon during our measurement period (*20*). There is also no clear correlation between wave height and timing of the flows (Fig. 6B).

Prolonged turbidity currents have previously been attributed to hyperpycnal river floods that plunge to form a turbidity current moving along the seafloor (*21*). However, the turbidity currents described here did not coincide with Congo River floods (Fig. 6B). In addition, the Congo River is characterized by particularly low sediment concentrations that would not allow the river water to plunge beneath the saline ocean water (*21*–*23*). However, previous cable breaks in the Congo Canyon show that powerful turbidity currents are more common during the months in which the Congo River discharge is elevated (*24*). This association suggests a connection between river discharge and turbidity current frequency, even if the turbidity currents are not formed directly by plunging river water. For example, it is possible that increased river discharge produces more rapid sediment deposition or large-scale switching of the lowermost branches of the braided river mouth, which may lead to more unstable slopes (*24*, *25*).

It has been previously shown that sustained turbidity currents may be initiated by prolonged slope failures called breaches. These sustained slope failures tend to occur in close-packed sands, which generate negative pore pressures once disturbed, resulting in a progressively up-slope migrating head scarp (*26*, *27*). However, previously observed breaches only lasted for up to several hours, rather than several days, as would be needed to trigger these weeklong flows in the Congo Canyon. It cannot be ruled out that more sustained breaching may occur in deep water, or prolonged slope failures may be triggered by other (as yet unknown) processes. However, it appears that sustained turbidity currents are unlikely to result from sustained sources such as plunging river floods or breaching. We submit that sustained turbidity currents may develop from a short-lived sediment source due to flow stretching, as a result of an erosive frontal-cell that outpaces and feeds an expanding trailing body.

### Comparisons with previous laboratory experiments

As outlined in Results, the structure of these turbidity currents in the Congo Canyon differs significantly from many laboratory-scale flows (Fig. 4). For example, in short-lived experimental flows (termed surges), the head does not outrun the body, and the body is rather poorly developed (Fig. 4A). This may be related to an inability for these relatively slow-moving and weak experimental flows to entrain sediment from the bed (*28*). Entrainment of seafloor sediment is needed to develop a self-sustaining head (that is, a frontal-cell) such that this initial self-sustaining part of the flow can both push aside the surrounding seawater and outrun the body. If the head has a similar or lower density than the body, then the body will tend to be faster than the head because it benefits from being in the lee of the head.

However, the laboratory experiments of Sequeiros *et al*. (*18*) show how the head of the flow may entrain sediment, thereby making the head self-sustaining. In these experiments, the head is seen to become denser and faster as it moves downslope, thereby meeting the criteria of self-acceleration (*29*). Sequeiros *et al*. (*18*) did not explicitly discuss the implications of a self-sustaining head for flow stretching, nor did their experiments create a prolonged trailing body. However, assuming that sediment in the trailing body does not settle out, a self-sustaining head will tend to run away from the body and thus stretch the flow.

### Comparison with previous measured oceanic turbidity currents

These flows in the Congo Canyon have significantly different durations and structures from previously measured oceanic turbidity currents (Fig. 2). We are aware of seven other shallower water locations where vertical profiles of velocity or concentration (that is, backscatter) have been measured in oceanic turbidity currents, in each case using ADCPs (*30*–*34*). In some cases, the ADCP velocity profiles were measured only every hour, and the flows were only a few hours in duration such that the frontal part of the event was probably not captured.

Turbidity currents in these other locations lasted for between a few minutes and ~10 hours (Fig. 2). These flows had a relatively consistent structure in which the maximum flow velocity occurred almost immediately behind the flow front, which was followed by a continuous decline in velocity (Fig. 2). This structure is similar to that seen in surge-like laboratory experiments (Fig. 4A). The first part of this structure also broadly resembles the frontal-cell within the Congo Canyon flows. However, turbidity currents at these other sites lack the elongated body that trails behind the frontal-cell in the Congo Canyon flows. Studies of flows in Monterey Canyon provide measurements from multiple locations that document flow durations at several points in the canyon (*31*, *34*). These flows are noteworthy because they did not stretch. Their duration initially decreased from 8 to 6 hours and then remained at 6 hours (*30*).

Here, we propose that flows in these other locations also comprise a fast-moving frontal-cell (Fig. 3B), which is erosive and thus self-sustaining. The frontal-cell also sheds sediment-laden fluid into the trailing flow. However, in locations other than the Congo Canyon, we propose that sediment shed into the trailing body tends to settle out rapidly, thereby counteracting flow stretching. If this is the case, flow stretching will be more pronounced in finer-grained turbidity currents in which sediment will settle more slowly.

This hypothesis is consistent with available field data indicating that flows in the Congo Canyon are significantly muddier than in the other seven locations. For example, deposits on the floor of Monterey Canyon typically comprise relatively coarse sand (*35*), whereas deposits on the canyon floor near our mooring sites in Congo Canyon consist of laminated sediments mainly composed of clay and silt (fig. S8). Deposits found on the lobe at the termination of the Congo canyon-channel system are also relatively fine-grained (*36*).

We therefore suggest that mud- and sand-rich turbidity currents tend to have different flow structures and durations. Mud-rich flows will have much better developed bodies, which may become self-sustaining and stretch. Sediment tends to settle out from the body of sand-rich flows so that their body is less well developed, and they primarily consist of a frontal-cell. Rapid settling from the body and tail of the sand-rich flows substantially reduces the degree to which these flows stretch.

### Wider implications of sustained turbidity currents

This study shows how flow stretching can generate prolonged and powerful turbidity currents, which flushed the Congo Canyon for ~33% of the 120-day measuring window in 2009–2010 (Fig. 3A). The wider implications of these prolonged flows of sediment and organic carbon into the deep ocean are profound. The Congo Canyon is directly connected to the Congo River, which drains about 2.2% of the Earth’s land surface, mostly covered by tropical vegetation. The total organic carbon transport through the submarine canyon can be estimated by assuming that, during 33% (Fig. 3A) of the time, turbidity currents are transporting an average of 2100 kg of sediment per second (Table 1) that comprises 3 to 5% of organic carbon (*37*). This transport results in the Congo Canyon carrying around 1.2 to 2.6% of the terrestrial organic carbon buried annually in the world’s oceans (*9*). These sustained turbidity currents favor transfer of organic carbon into the deep sea (*8*), and they help to explain widespread oxygen deficits linked to carbon remineralization (*37*). Sustained input and degradation of large amounts of organic matter have strongly affected ecosystem functioning in the deep sea. Unusual seafloor communities have recently been described across the distal Congo Fan, resembling those based on chemosynthesis at cold seep sites (*38*).

The powerful and sustained nature of the turbidity currents described here illustrates the challenge of mitigating hazards to important seafloor infrastructure, which underpins global telecommunications and energy supplies (*5*–*7*, *39*). Previous attempts to mitigate this hazard include work to reroute a major gas pipeline beneath the Congo Canyon, using directional sub-seafloor drilling (*39*). Here, we provide new insights into the duration, velocity, and density structure of turbidity currents, which are crucial for determining impact forces on seabed infrastructure. We show that subsea cables and pipelines must withstand powerful flows with discharges comparable to the Congo River or Mississippi River that persist for nearly a week rather than for only hours or minutes.

## MATERIALS AND METHODS

### ADCP data collection

ADCP data were collected during two periods (*5*, *6*). From December 2009 to March 2010, a 300-kHz ADCP was suspended 85 m above the Congo Canyon floor from a fixed mooring. The second ADCP was located on a mooring ~700 m down-canyon and operated at a lower frequency of 75 kHz (*5*). These moorings were located on the channel floor at a water depth of ~2000 m (Fig. 1C) (*5*). Two 300-kHz ADCPs were subsequently deployed at the 2013a and 2013b sites (Fig. 1B) from January to March 2013, suspended 66 m above the canyon floor (*6*). The 2013b site was located 22 km down-canyon from the 2013a site (Fig. 1B).

An ADCP measures flow velocity by transmitting four beams of acoustic energy into the water column (*40*), which were set at 20° to the vertical, and at 90° to each other. The Doppler shift of the backscattered signal from within the flow was recorded over time and used to calculate the along-beam velocity for each of the individual beams at different vertical intervals (bins) throughout the flow. A velocity vector was then reconstructed by combining the along-beam velocities with the heading and tilt information recorded by the ADCP. The ADCPs were set up to measure a three-dimensional velocity profile that consisted of 39 individual measurements (bins), with a vertical spacing of 2 m. A velocity profile was recorded every 5 s. Figure 3A shows velocities that have been averaged over a period of 500 s.

The single ADCP used in 2009–2010 recorded flow velocities and acoustic backscatter to within 3 m of the bed and was located near the center of the canyon floor thalweg (*5*). However, the two ADCPs in the January-March deployment of 2013 were unable to record data within the lowermost 18 m of the flows (*6*). This was because the moorings were located toward the margins of the canyon floor such that one or more of the ADCP’s four beams returned strong echoes from steeply dipping canyon flanks. These strong returns prevented data acquisition within the lower 18 m of flow (*6*). Detailed analysis of distances to seafloor returns from individual beams indicates that at both 2013 sites, the ADCPs were located above the flat canyon thalweg but within ~45 m of the canyon wall (fig. S1).

### Flow front velocity derived from four ADCP beams

The front velocity of the turbidity current is derived from the difference in arrival time of flow at each of the four acoustic beams (fig. S2). The footprint of four ADCP beams spans ~45 m × 45 m on the canyon floor. The arrival time of the flow at each of the beams is recognized by a strong increase in the backscatter intensity (fig. S2C). The distance between the centers of the four beams is known, but their orientation relative to the direction of flow front propagation is uncertain (angle α in fig. S2B). The distances between centers of ADCP beams must therefore be calculated in the direction of travel of the flow front (fig. S2B) to calculate flow front velocities. This is done by expressing the arrival time at the individual beams as a function of the flow front velocity. The resulting frontal flow velocity was found to be 1.2 to 1.5 m/s. Uncertainties in the calculated flow front velocity are due to uncertainties in the arrival times resulting from the 5-s spacing between consecutive measurements (fig. S2). This method assumes that the ADCP heading is stable over the period considered (a few tens of seconds, fig. S2C), that the frontal velocity does not fluctuate significantly as it travels between ADCP beams, and that the flow front is reasonably straight over distances of several tens of meters orthogonal to its direction of travel.

### Extracting velocity fluctuations

Large-scale velocity fluctuations were used here as a proxy for turbulence intensity within the flow (Fig. 3C). These velocity fluctuations were calculated by (i) calculating the average velocity over a 1-min period, (ii) subtracting the 1-min average velocity from a single velocity measurement made at the center of this 1-min period, (iii) squaring the velocity difference values, (iv) calculating an average velocity difference profile over a 500-s period, and (v) taking the square root of the resulting average. Given the 5-s measuring resolution and the 1-min moving average, these velocity fluctuations are biased toward large-scale velocity fluctuations and should therefore only be used as a crude indicator of turbulence intensity. In addition, it should be taken into consideration that the velocity fluctuations will also include any possible noise on the data.

### Sediment concentration from ADCP backscatter

Suspended sediment concentrations within the flow (Fig. 3D) were obtained by inversion of the acoustic backscatter acquired by a 300-kHz ADCP (fig. S3) (*41*). They were validated using a second ADCP located on a mooring ~700-m down-canyon, which operated at a lower frequency of 75 kHz (*5*). This inversion analysis is innovative, because these are the first sediment concentration values derived for any full-scale turbidity current from multiple-frequency ADCPs.

The basic approach is to first convert the raw backscatter data from the receiver signal strength indication (RSSI) units to a logarithmic decibel scale and then compensate for any bias due to variable orientation of the ADCP beams during the measurements. Using the 300-kHz ADCP, the loss of strength (attenuation) of echo from the bed was calculated by comparing the bed echo strength before the flow event to the echo strength during the flow. A sediment concentration profile was inferred assuming uniform grain size throughout the flow, along with a reference sediment concentration just above the bed. The sediment concentration profile within the flow was computed in an iterative fashion such that it best fit the observed change in attenuation of the bed echo before and during the flow. We then used the second, 75-kHz ADCP, data to constrain the most likely grain size within the flow at each given point in time. This assumes that there is a single grain size in a vertical profile above the bed at each given point in time. This is done by taking the sediment concentration profile and reference concentration inferred previously from the 300-kHz ADCP data and determining the grain size(s) that produces the closest fit to the bed attenuation seen in the 75-kHz ADCP data. Finally, we were able to assess assumptions regarding a uniform grain size within the flow using a calculated parameter, *K*_t_, which essentially indicates local departures from the backscatter profiles assuming a single grain size.

This method, which first used the 300-kHz ADCP data and then the additional 75-kHz ADCP data, is now outlined in more detail below. The raw backscatter data in RSSI, *E*, were converted to linear backscatter counts, *V*, for all beams using

$$V=10^{K_{c}(\frac{\left(E-N\right)}{20})}$$ (1)

where *K*_c_ is a measured constant for each of the four transducers (values supplied for deployed instruments by Teledyne RDI Inc.) and *N* is the noise level for each transducer channel, determined as the mean of the acquired values at the maximum sediment attenuation during the acquisition period. The orientation of the ADCP biased the measured backscatter intensity, probably due to the sidelobe interference in the near-bed bins and variation in the bed elevation across the interrogation volume. Hence, for each set of four beam profiles acquired with the 300-kHz ADCP, the heading of the ADCP compass was used to remove the heading bias in backscatter intensity. This allowed us to recover and use the beam that had the lowest acoustic sidelobe interference. Backscatter (*V*) from homogeneous suspensions of sediment is randomly distributed, so profiles were averaged by determining the root mean square value of 100 consecutive profiles over 500 s.

The ADCP measurement bin with the consistently highest raw echo magnitude throughout the record (bin 40) was assumed to contain a bed echo. The bed echo attenuation throughout the duration of the turbidity current was then calculated as the ratio of the backscatter in bin 40 during the event to the backscatter in the same bin of the same transducer beam at the same compass heading before the event. Figure S4 shows the mean bed attenuation values averaged for the four transducers for the duration of the event, as well as the bed attenuation of the lower-frequency 75-kHz ADCP. The values are expressed in decibel and are derived as 20log_10_(*A*_bed_), where

$$A_{\text{bed}}=20\;{\text{log}}_{10}(\frac{V_{\text{event}}}{V_{\text{clear water}}})$$ (2)

An initial sediment concentration profile can then be derived using water column backscatter and an initial reference mass concentration at the bed, assuming that the flow contains a uniform grain size. This is because the mass concentration of suspended sediment, *M*(*r*), as a function of range from the ADCP transducers, *r*, is related to the backscatter magnitude, *V*_rms_(*r*), by the following relationship (*42*)

$$M(r)={\left(\frac{V_{\text{rms}}(r)\phi (r)r}{K_{t}K_{s}(r)}\right)}^{2}e^{4(\alpha_{w}r+\alpha_{s}(r))}$$ (3)

where φ(*r*) is a correction for the transducer’s near field (*43*); *K*_t_ should be constant and describes the sensitivity of the individual transducer; *K*_s_ is related to the scattering properties of the sediment in suspension and is a function of the particle grain type and size relative to the acoustic frequency; α_w_ is the sound attenuation due to the properties of the water and is calculated using the formula of Francois and Garrison (*44*, *45*) as 0.0079 nepers/m, using a measured mean water temperature of 3.7°C and mean depth of 1924 m, with an assumed pH of 8 and 35 ppt (parts per thousand) salinity; and α_s_ is the sound attenuation due to suspended sediment. The range, *r*, is divided into discrete units corresponding to the bin size of 2.13 m along the acoustic beams, which are inclined at 20° to the vertical and correspond to a 2.0-m vertical bin spacing through the water column.

Solving Eq. 4 is nontrivial because the α_s_(*r*) expression is itself a function of *M*(*r*)

$$\alpha_{s}(r)=\int_{0}^{r}\xi (r)M(r)dr$$ (4)

where ξ(*r*) is a function of the particle type and size relative to the acoustic frequency. The explicit solution of Lee and Hanes (*46*), which assumes that the grain size is constant for all ranges from the ADCP transducers, was used to close the equations. This assumption of a uniform grain size is necessary given the lack of information about grain size variability, but it also removes the requirement of knowing the values of the *K*_s_ and *K*_t_ constants in Eq. 4 (*47*). As a result, the sediment concentration profile *M*(*r*) is given by

$$M(r)=\frac{\beta {\left(r\right)}^{2}}{\beta_{\text{ref}}^{2}/M_{\text{ref}}-4\xi \int_{r_{\text{ref}}}^{r}\beta {\left(r\right)}^{2}dr}$$ (5)

where

$$\beta (r)=V_{\text{rms}}(r)re^{2\alpha_{w}r}$$ (6)

and *M*_ref_ is a known concentration at a reference range, *r*_ref_. The first value of *M*_ref_ used to determine *M*(*r*) is a guess, because the concentration at the reference range is unknown. However, for the inversion to remain constrained, the reference range needs to be set at the farthest range, that is, the bed, to prevent the accumulation of errors beyond the reference range. The attenuation coefficient constant, ξ, for the single, assumed, grain size is derived as the sum of acoustic scattering and viscous absorption expressions through the water column. The acoustic scattering component is evaluated by first calculating the scattering cross section, χ, using the heuristic expression of Moate and Thorne (*48*), which was developed as a generic expression for sands of varying mineralogy. The scattering attenuation coefficient is then determined by

$$\xi_{\text{scattering}}=\frac{3\chi }{4a}$$ (7)

The viscous absorption component, ξ_viscous_, is calculated using Urick’s (*49*) formula using a value of 1.52 m^2^/s for the kinematic viscosity of water at 3.7°C and an assumed density of 2650 kg/m^3^ for the sediment. The plot in fig. S5 shows the values of the sediment attenuation coefficient, ξ, for a single value of grain diameter across the range of 0.001 to 1 mm for both ADCP frequencies. For small particles, the viscous absorption term dominates and is at a peak for clay/silt particles. For diameters greater than ~400 μm, the scattering term dominates and ξ increases with diameter. The cumulative through-water attenuation of the derived mass concentration profile can be calculated from the transducers to the bed (bins 1 to 39), *A*_profile_, using the profile of *M*(*r*) obtained in Eq. 5

$$A_{\text{profile}}=e^{\int_{o}^{r_{\text{ref}}}\;-4\xi M(r)dr}$$ (8)

The reference mass concentration can then be adjusted iteratively through the above equation set until the cumulative attenuation of the derived concentration profile matches the bed echo attenuation and, in essence, when the difference between the two attenuation values, *A*_profile_ − *A*_bed_, reduces to approximately zero.

The second 75-kHz ADCP acquired backscatter data over a range of ~230 m above the bed at a location on the canyon floor approximately 700 m downstream from the 300-kHz ADCP. The bed echo attenuation, *A*_bed_ (fig. S6), enables a method of estimating the grain size in suspension by evaluating *A*_profile_ at 75 kHz using the *M*(*r*) derived with the 300-kHz ADCP. This assumes that the suspended sediment structure of the flow remains unchanged over the distance of ~700 m along the thalweg. *A*_bed_ − *A*_profile_ is therefore calculated for all profiles for a single grain size.

The suspended grain size for a given concentration profile is found by iterating through a range of possible grain sizes and comparing the error between *A*_bed_ and *A*_profile_ at 75 kHz for each profile. Two solutions were found for each set of profiles, considering a plausible range of grain diameters from 0.1 to 1000 μm. The values of *A*_bed_ − *A*_profile_ for a single averaged profile are shown in fig. S6, and the two grain size solutions throughout the event are displayed in fig. S7.

The smaller of the two possible grain sizes (4.23 μm) is the most realistic option, because it closely matches the average *D*_50_ of nearby sediment cores that was 2.8 μm (fig. S8). The final result (fig. S9) was therefore derived using a uniform grain size of 4.23 μm throughout the event.

A key assumption is that the flow contains a single grain size, and we note that grain size may vary vertically and through time, and multiple grain sizes may be present at one location. In general, sediment concentrations tend to be higher in the lower part of the flow, as expected for stable density stratification (fig. S9). The highest sediment concentrations occur at the base of the frontal-cell. Sediment concentrations also increase in the tail of the flow, where vertical concentration gradients likely become stronger. However, there appear to be artefacts within this sediment concentration data set (shown in red circles in fig. S9), where higher sediment concentrations overlie lower sediment concentrations. This inverted density stratification is likely to be unstable and unrealistic. Our analysis assumes a single grain size within the flow, and these artefacts may be due to vertical changes in grain size or other factors such as zones of strong turbulence or the influence on acoustic returns of refraction at interfaces with different densities.

To provide an estimate of uncertainties in the calculated sediment concentrations, and the amounts of sediment transported by each turbidity current (Table 1), we also calculated sediment concentrations for flows with uniform grain size that vary from 3 to 20 μm, not just 4.23 μm. This grain size range reflects modal grain sizes seen in canyon floor cores in the general vicinity of our mooring site (fig. S8). Flows containing a uniform grain size of 3 μm would have sediment concentrations that are 90% of those calculated using a uniform grain size of 4.23 μm. Conversely, flows with a uniform grain size of 20 μm would have sediment concentrations that are 320% of the values calculated using a grain size of 4.23 μm. These calculations, with uniform sediment grain sizes of 3 to 20 μm, are then used to give the range of sediment transported by an average turbidity current (for example, ~1.1 to 3.8 Mt) in the text. However, these ranges neglect the effects on backscatter inversions from spatial or temporal changes in grain size within a single flow.

As a test of the methodology, for a concentration profile, *M*(*r*), a calibration constant, *K*_t_, can be derived for all ranges, *r*, by evaluating

$$K_{t}=\beta M^{-1/2}e^{2r\alpha_{s}}$$ (9)

If the uniform grain size assumption for a given profile is true, then the value of *K*_t_ should remain constant throughout the range from the transducers to the bed, because *K*_t_ is a fixed acoustic property of the transducer. The values of *K*_t_ are plotted in fig. S10A. During the first 20 to 30 min, *K*_t_ increases significantly in the near-bed region (fig. S10B). This suggests that the mean grain size increases toward the bed and that, as a result, sediment concentrations in those initial near-bed bins are probably underestimated in fig. S9. Throughout the remainder of the event, the profiles of *K*_t_ remain approximately constant, suggesting that the grain size remains relatively constant, albeit with some minor variations that map to the artefacts discussed above (red circles in fig. S9).

### Bed shear stresses and Shield’s diagram

Bed shear stresses determine which sediment sizes the flow can transport, and whether the flow will pick up additional sediment from the bed or sediment will settle out of the flow. Bed shear stresses are expressed as a bed shear velocity (u_*_), which can be calculated in two ways.

First, u_*_ can be derived from the shape of the vertical velocity profile below the velocity maximum (*50*). This method assumes that the velocity profile near the bed is logarithmic. The slope of that log-linear plot is used to calculate the value of u_*_. We only used velocity profiles that had three or more measured values below the velocity maximum. In some parts of the flow, the ADCP could not measure below the velocity maximum, and this method cannot be applied. These u_*_ values are plotted as blue squares in fig. S11A.

The second method of calculating the bed drag coefficient links the bed shear velocity to the maximum flow velocity (*51*). The bed drag coefficient is assumed to only relate to bed roughness and will be constant for a fixed location during the flow. This allows us to extrapolate bed shear velocities for the whole flow, based on the maximum flow velocities. Bed shear velocities are then used to calculate Shields numbers and boundary Reynolds numbers for different grain sizes. We show calculations for three grain sizes: 200, 80, and 10 μm. By plotting the results in a Shields diagram (fig. S11B) (*28*), it can be seen that the frontal-cell is powerful enough to suspend all three grain sizes under the assumption of a cohesionless bed. The decreasing values of the Shields number and the boundary Reynolds number over the body of the flow indicate that erosion will become less vigorous, but sediment will remain in suspension. Lower values of u_*_ in the tail show that sediment is no longer supported and will begin to settle out. It appears that this produces relatively high sediment concentration layers near the bed within the tail of the flow (fig. S9).

### Calculations of water, sediment, and organic carbon discharges

Water discharges were calculated by multiplying the flow velocity measured by the ADCP at each height above the canyon floor (that is, each bin) by the width of the canyon at that corresponding height and the vertical height range of each bin (~2 m). These values were then summed to give the total water discharge through time (fig. S12A).

Estimates of sediment concentration were then derived from ADCP measurements at each bin, as described above. These sediment concentrations will have significantly greater uncertainties than the ADCP’s velocity measurements (see discussion above). Sediment concentrations were multiplied with water discharges calculated for each bin to calculate the overall sediment flux during each turbidity current (fig. S12B). This sediment flux may be an underestimate because it does not include the lower 3 to 4 m of the flow, which is within the ADCP blanking distance. The average sediment flux during a turbidity current is estimated to be 2.1 × 10^3^ kg/s (Table 1), assuming a uniform grain size of 4.23 μm. Assuming a uniform grain size within each flow, which varies between 3 and 20 μm, the average amount of sediment carried by an individual turbidity current then becomes 2.1 × 10^3^ to 6.7 × 10^3^ kg/s. The average turbidity current in the Congo Canyon would then carry a total of ~1.2 Mt of sediment during its 6.7-day duration, assuming a uniform grain size of 4.23 μm, or 1.1 to 3.8 Mt for uniform grain sizes that range between 3 and 20 μm. The amount of sediment transported by turbidity currents each year (~22 Mt) is calculated by assuming that the average sediment flux of 2.1 × 10^3^ kg/s occurs during 33% of the time (Fig. 3A).

An average organic carbon content of 3 to 5% weight was assumed for the sediment carried within the turbidity current. This value is based on the organic carbon content measured within turbidity current deposits on the Congo Fan, which are dominated by terrestrial (rather than marine) organic carbon (*37*). This assumes that the composition of sediment within the flow is broadly similar to that buried in flow deposits. Oxidization of organic carbon during burial of these sediments will result in the amount of organic carbon within the flow being underestimated. This calculation results in an annual flux of 0.5 to 1.1 Mt/year of predominantly terrestrial organic carbon through the canyon. This is 1.2 to 2.6% of the estimated 43 Mt of terrestrial organic carbon that is buried globally in the oceans each year (*9*).

### Congo Canyon turbidity current triggering

To better understand potential triggers, the timing of turbidity currents in the Congo Canyon was compared to environmental variables such as river discharge and wave heights. We were unable to carry out robust statistical analysis of turbidity current triggering due to the small number (six events) of observed turbidity currents. Our analysis of how these flows were triggered therefore remains qualitative.

Turbidity current triggering has previously been linked with sediment-laden river flood water discharge at the coast (*21*). To test this relationship, turbidity current timing in the Congo Canyon was compared to river discharge data from the Kinshasa gauging station (4.3°S, 15.3°E). This gauging station is located ~450 km upstream from the mouth of the Congo River, and it is the closest available station.

Large waves, internal tides, and standing waves are thought to be able to trigger turbidity currents in submarine canyons (*51*–*53*). Ocean buoys can provide an excellent record of these variables, but their global coverage is spatially variable and these data are not always freely available. For the Congo Canyon, we therefore used global model data. Six-hour estimates of significant wave height (in meters), mean wave period (in seconds), mean wave direction (in degrees), and surface pressure (in pascal) were obtained from the ERA-Interim global atmospheric reanalysis model produced by the European Centre for Medium-Range Weather Forecasts (ECMWF) (*54*) at the head of the Congo Canyon (fig. S13). The data were gridded at a resolution of 0.125°. The data revealed no clear relationship between ocean wave or surface pressure characteristics and turbidity current frequency (fig. S13). Turbidity current occurrence does not coincide with peaks in significant wave height, nor do magnitudes vary to the extent that they have when previously linked to turbidity current triggering (*55*, *56*). It is therefore unlikely that the measured turbidity currents were triggered either directly by surface waves or after a delay as a consequence of wave action on the seafloor.

Earthquake records during the period in which ADCPs were deployed were obtained from the U.S. Geological Survey Advanced National Seismic System Comprehensive Earthquake Catalog (ComCat; https://earthquake.usgs.gov/earthquakes/search/). The timing of earthquakes (*M*_w_ > 2.5) within 300 km of the head of the Congo Canyon was compared to known turbidity current arrival times.

## Supplementary Material

http://advances.sciencemag.org/cgi/content/full/3/10/e1700200/DC1

## SUPPLEMENTARY MATERIALS

Supplementary material for this article is available at http://advances.sciencemag.org/cgi/content/full/3/10/e1700200/DC1 fig. S1. Distance to the seafloor in different directions measured by an individual ADCP beam at the up-canyon 2013a site and the down-canyon 2013b site in 2013. fig. S2. Illustration of the method used to calculate flow front velocity. fig. S3. Raw backscatter plot. fig. S4. The bed echo attenuation for 300- and 75-kHz ADCP during the turbidity current. fig. S5. Sediment attenuation coefficient (ξ) for 300- and 75-kHz frequencies, by particles with diameters between 1 and 1000 μm. fig. S6. Difference between the bed echo attenuation (*A*_bed_) and the predicted cumulative echo attenuation (*A*_profile_) within the water column from the 75-kHz ADCP data. fig. S7. The suspended grain size results derived from the comparison between the 75-kHz ADCP bed echo. fig. S8. Cores from floor of Congo Canyon. fig. S9. Sediment concentration (g/liter) derived using the ADCP backscatter magnitudes. fig. S10. The calibration constant *K*_t_. fig. S11. Bed shear stresses generated by the flow. fig. S12. Comparisons of the instantaneous sediment and water discharges in the Congo Canyon turbidity current shown in Fig. 2, with the mean annual discharges of water and sediment in major rivers. fig. S13. Comparison of turbidity current arrival times with possible triggering factors in the Congo Canyon. fig. S14. Increase in flow duration caused by flow stretching, which is due to a difference in the speed of the front and tail of the flow. table S1. Flow durations, thicknesses, and peak velocity measured at heights in excess of 18 m above the bed in 2013.
