# Supplementary material for: Newly recognized turbidity current structure can explain prolonged flushing of submarine canyons
Source: Sci Adv. 2017 Oct 4;3(10):e1700200. doi: 10.1126/sciadv.1700200 (PMC5627984; doi:10.1126/sciadv.1700200)
Supplement: http://advances.sciencemag.org/cgi/content/full/3/10/e1700200/DC1 [file supp_3_10_e1700200__index.html]

Science Advances | Science Advances

## Supplementary Materials

**This PDF file includes:**

- fig. S1. Distance to the seafloor in different directions measured by an individual ADCP beam at the up-canyon 2013a site and the down-canyon 2013b site in 2013.
- fig. S2. Illustration of the method used to calculate flow front velocity.
- fig. S3. Raw backscatter plot.
- fig. S4. The bed echo attenuation for 300- and 75-kHz ADCP during the turbidity current.
- fig. S5. Sediment attenuation coefficient (ξ) for 300- and 75-kHz frequencies, by particles with diameters between 1 and 1000 μm.
- fig. S6. Difference between the bed echo attenuation (*A*bed) and the predicted cumulative echo attenuation (*A*profile) within the water column from the 75-kHz ADCP data.
- fig. S7. The suspended grain size results derived from the comparison between the 75-kHz ADCP bed echo.
- fig. S8. Cores from floor of Congo Canyon.
- fig. S9. Sediment concentration (g/liter) derived using the ADCP backscatter magnitudes.
- fig. S10. The calibration constant *K*t.
- fig. S11. Bed shear stresses generated by the flow.
- fig. S12. Comparisons of the instantaneous sediment and water discharges in the Congo Canyon turbidity current shown in Fig. 2, with the mean annual discharges of water and sediment in major rivers.
- fig. S13. Comparison of turbidity current arrival times with possible triggering factors in the Congo Canyon.
- fig. S14. Increase in flow duration caused by flow stretching, which is due to a difference in the speed of the front and tail of the flow.
- table S1. Flow durations, thicknesses, and peak velocity measured at heights in excess of 18 m above the bed in 2013.

Download PDF

**Files in this Data Supplement:**

- Adobe PDF - 1700200\_SM.pdf
