# Supplementary material for: Newly recognized turbidity current structure can explain prolonged flushing of submarine canyons
Source: Sci Adv. 2017 Oct 4;3(10):e1700200. doi: 10.1126/sciadv.1700200 (PMC5627984; doi:10.1126/sciadv.1700200)
Supplement: http://advances.sciencemag.org/cgi/content/full/3/10/e1700200/DC1 [file 1700200_SM.pdf]

Science Advances

AAAS

advances.sciencemag.org/cgi/content/full/3/10/e1700200/DC1

Supplementary Materials for

Newly recognized turbidity current structure can explain prolonged flushing of submarine canyons

Maria Azpiroz-Zabala, Matthieu J. B. Cartigny, Peter J. Talling, Daniel R. Parsons, Esther J. Sumner, Michael A. Clare, Stephen M. Simmons, Cortis Cooper, Ed L. Pope

Published 4 October 2017, *Sci. Adv.* **3**, e1700200 (2017)
DOI: 10.1126/sciadv.1700200

This PDF file includes:

- fig. S1. Distance to the seafloor in different directions measured by an individual ADCP beam at the up-canyon 2013a site and the down-canyon 2013b site in 2013.
- fig. S2. Illustration of the method used to calculate flow front velocity.
- fig. S3. Raw backscatter plot.
- fig. S4. The bed echo attenuation for 300- and 75-kHz ADCP during the turbidity current.
- fig. S5. Sediment attenuation coefficient (ξ) for 300- and 75-kHz frequencies, by particles with diameters between 1 and 1000 μm.
- fig. S6. Difference between the bed echo attenuation (A_{bed}) and the predicted cumulative echo attenuation ($A_{profile}$) within the water column from the 75-kHz ADCP data.
- fig. S7. The suspended grain size results derived from the comparison between the 75-kHz ADCP bed echo.
- fig. S8. Cores from floor of Congo Canyon.
- fig. S9. Sediment concentration (g/liter) derived using the ADCP backscatter magnitudes.
- fig. S10. The calibration constant K_t.
- fig. S11. Bed shear stresses generated by the flow.
- fig. S12. Comparisons of the instantaneous sediment and water discharges in the Congo Canyon turbidity current shown in Fig. 2, with the mean annual discharges of water and sediment in major rivers.
- fig. S13. Comparison of turbidity current arrival times with possible triggering factors in the Congo Canyon.

- fig. S14. Increase in flow duration caused by flow stretching, which is due to a difference in the speed of the front and tail of the flow.
- table S1. Flow durations, thicknesses, and peak velocity measured at heights in excess of 18 m above the bed in 2013.

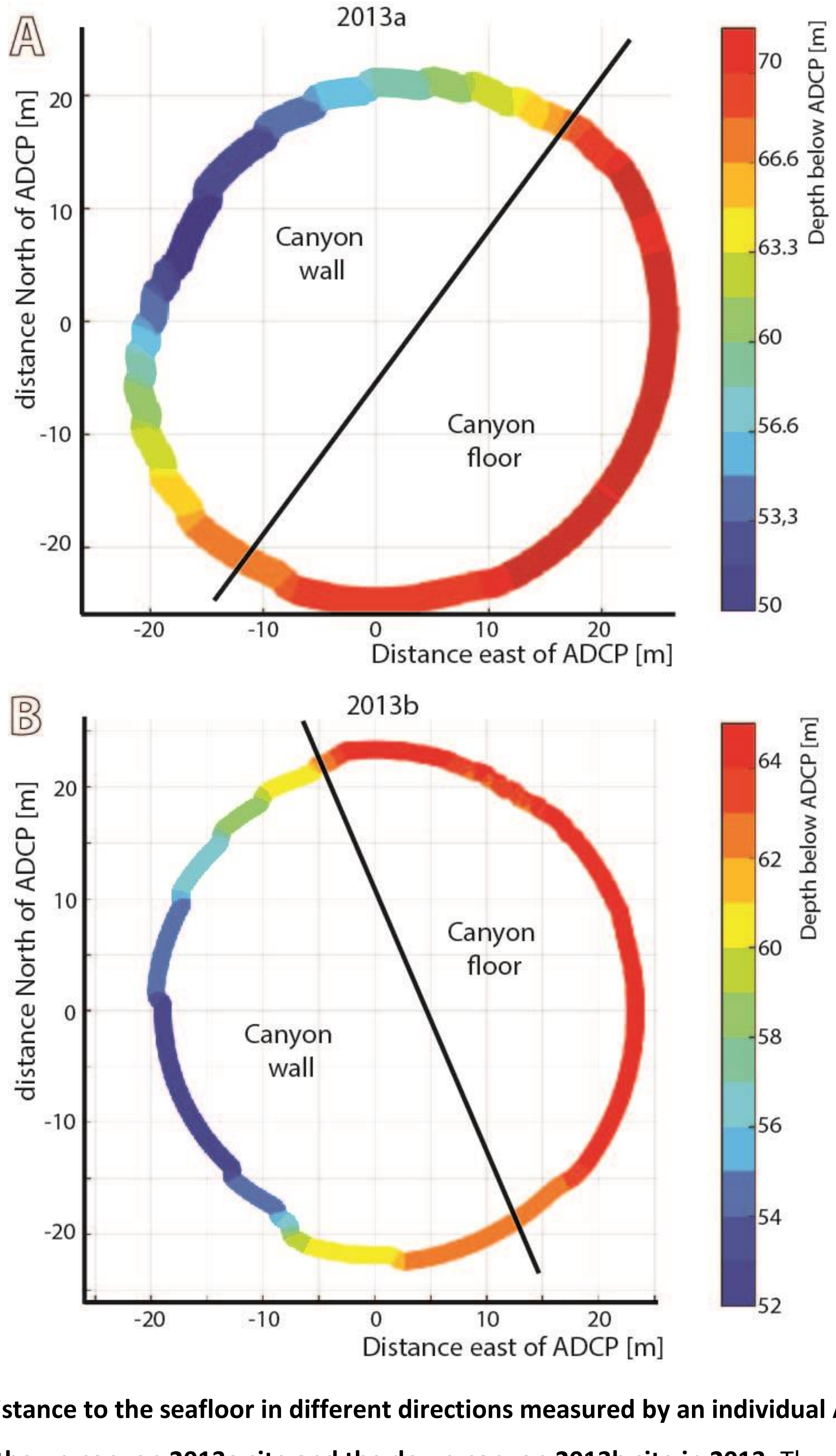

fig. S1. Distance to the seafloor in different directions measured by an individual ADCP beam at the up-canyon 2013a site and the down-canyon 2013b site in 2013. The centre of the ADCP beam's acoustic footprint is located on the margin of the flat (red colours) canyon floor and the more steeply inclined canyon margin (yellow-to-blue colours).

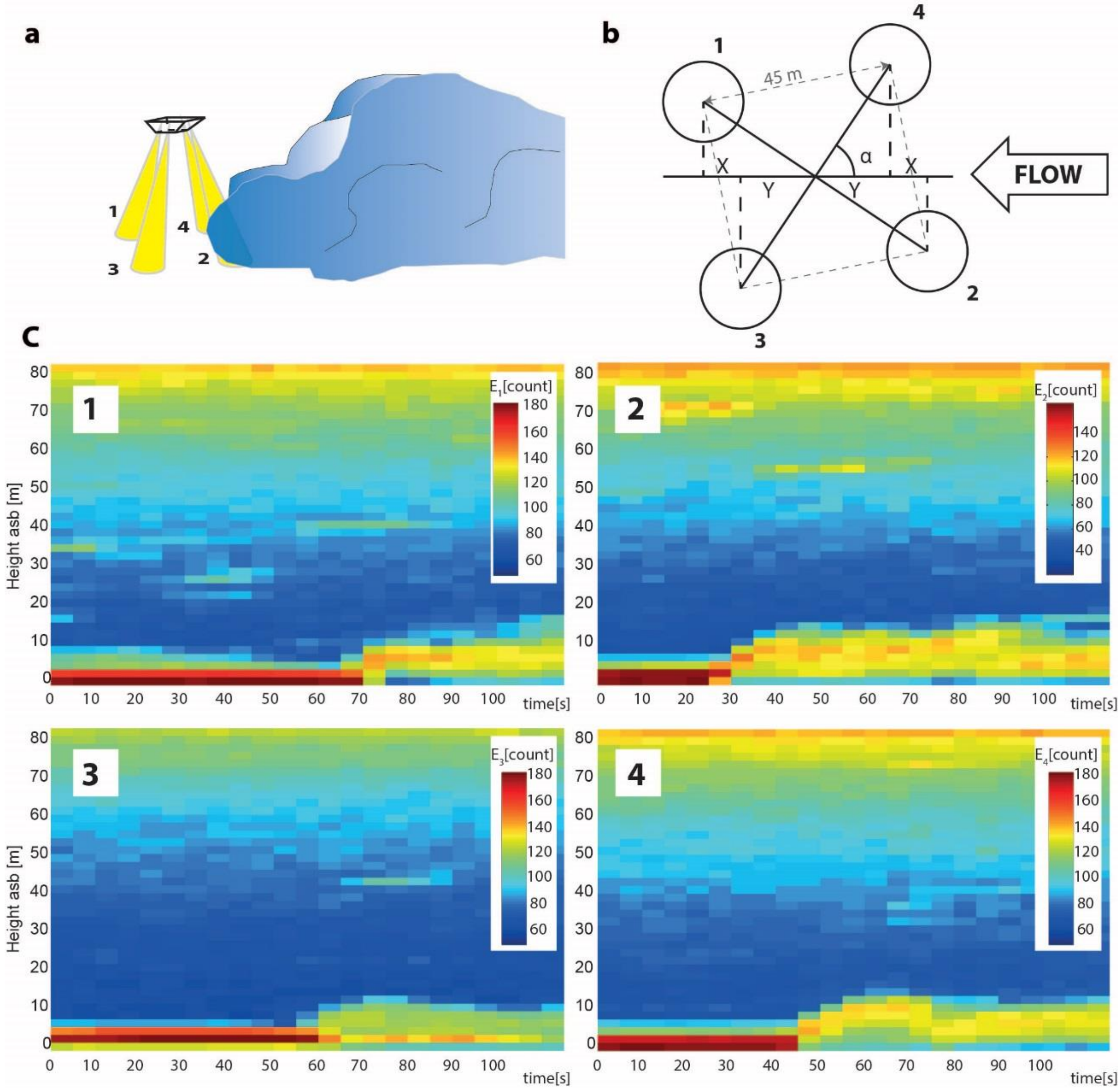

fig. S2. Illustration of the method used to calculate flow front velocity. (**A**) Schematic diagram of flow approaching the deployed ADCP. (**B**) The geometry of the four beams, which can be used to define the following relationships for flow front velocity (V) in terms of distances X and Y, and time delays between the flow from reaching ADCP beams 1 to 4 ($t_{4\text{-}2}$ for time between beams 4 and 2 etc); $V = X(t_4-t_2)= X(t_1-t_3)$; $V = 2Y (t_3-t_4)$; $V = (X+2Y)/(t_3-t_2) = (X-2Y)/(t_1-t_4)$. (**C**) Echo intensity (E; expressed in counts) recorded by each individual ADCP beam at flow arrival (yellow-greenish area). Highest echo intesity centered on 0 metre above seafloor (asb) indicates the position of the seabed. Number in each subpanel indicates the ADCP beam number.

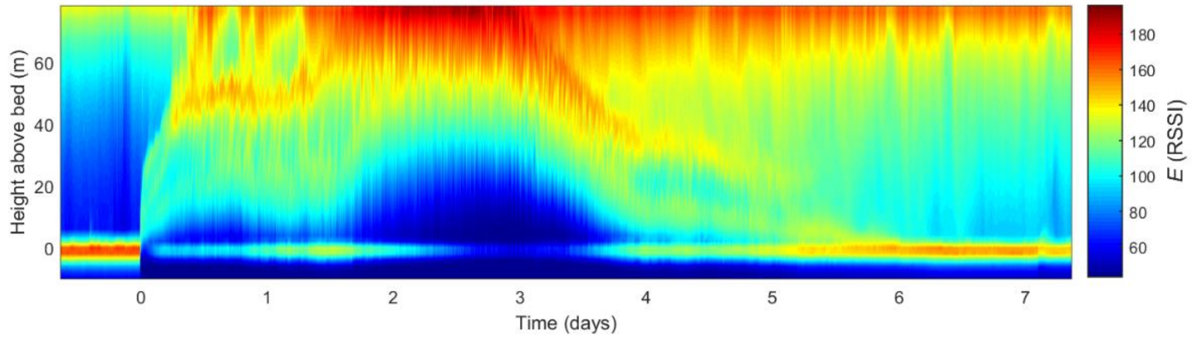

fig. S3. Raw backscatter plot. Backscatter data averaged over 500 seconds and all four beams of the 300 kHz ADCP throughout the turbidity current. The echo from the bed occurs around a height of 0 metre.

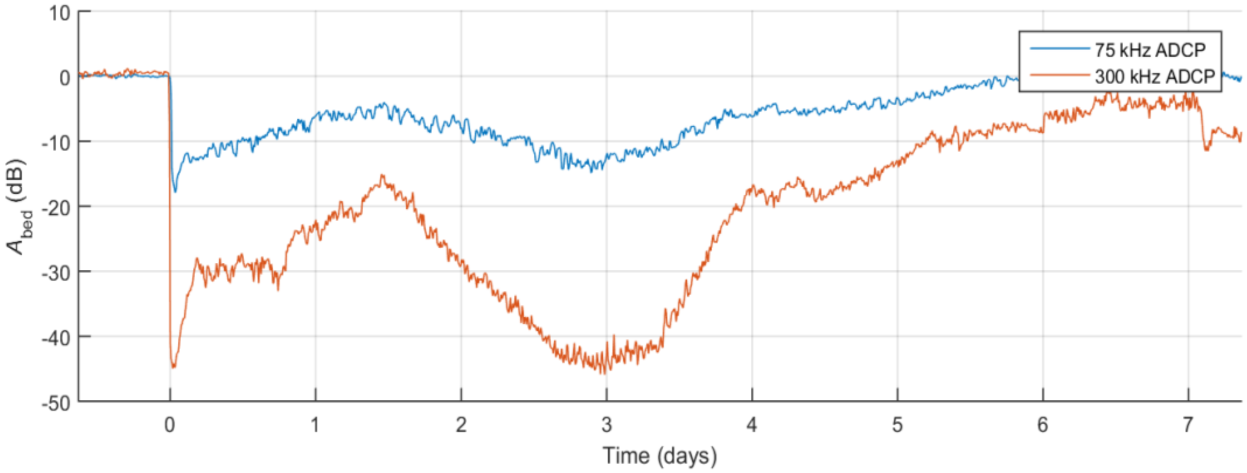

fig. S4. The bed echo attenuation for 300- and 75-kHz ADCP during the turbidity current.

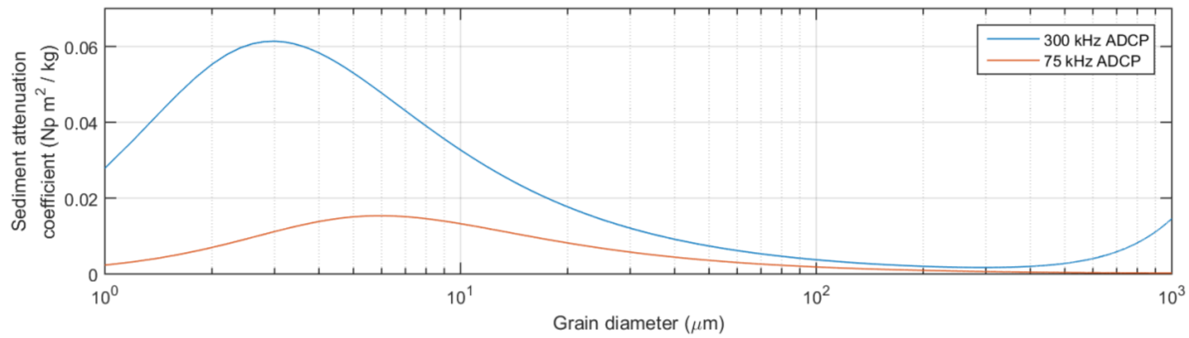

fig. S5. Sediment attenuation coefficient (ξ) for 300- and 75-kHz frequencies, by particles with diameters between 1 and 1000 μm.

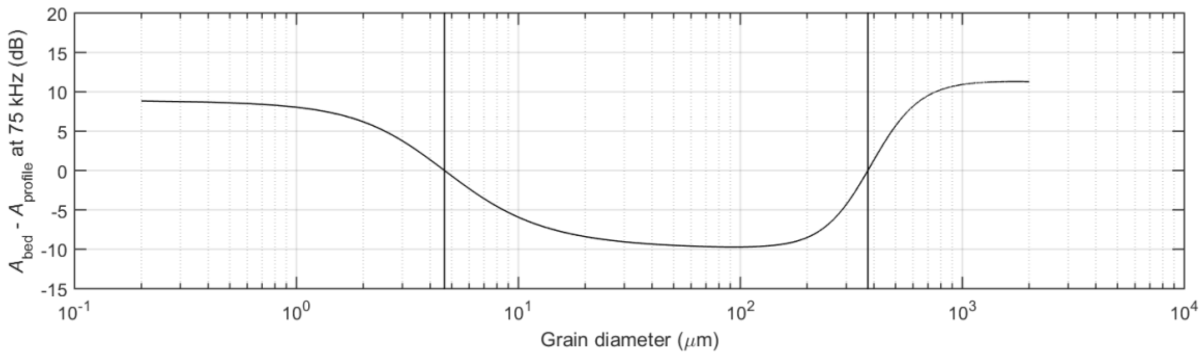

fig. S6. Difference between the bed echo attenuation (*A_{bed}*) and the predicted cumulative echo attenuation (*$A_{profile}$*) within the water column from the 75-kHz ADCP data. The value is $A_{profile}$ is based on the sediment concentration profile previously calculated from the 300 kHz ADCP data. The difference (A_{bed} – $A_{profile}$) is calculated for a range of grain sizes from 2 µm to 2000 µm. Only two grain sizes (shown by vertical lines) produce a zero value of A_{bed} – $A_{profile}$ and thus satisfy both the 75 kHz and 300 kHz backscatter observations. This analysis uses backscatter data comprising averages of 100 consecutive measurements (pings) acquired over 500 s. It also assumes that a vertical profile through the flow at a given point in time comprises only one grain size, whilst it is likely that grain size varies with height above the bed.

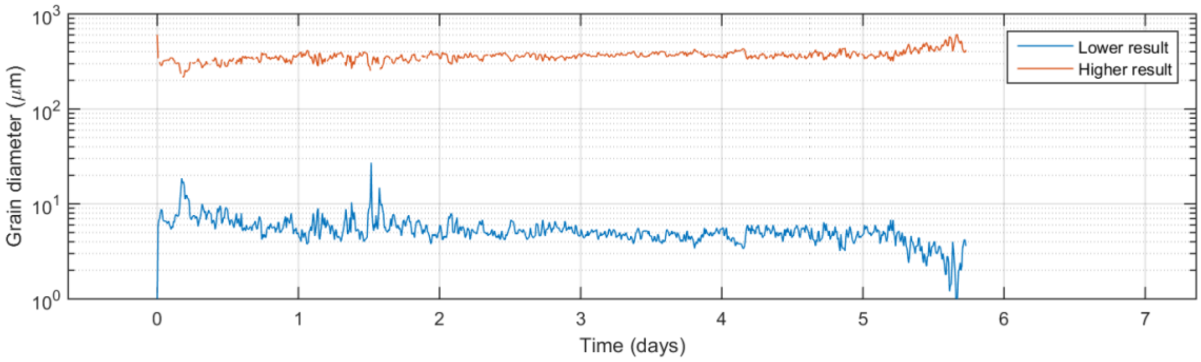

fig. S7. The suspended grain size results derived from the comparison between the 75-kHz ADCP bed echo. Two grain sizes satisfy both the 75 kHz and 300 kHz ADCP data ('lower result and higher result'; see fig. S6). This figure shows how those two grain sizes vary over time at the study site. However, this still assumes that there is a single grain size in the flow at each point in time, and that grain size is not changing with height in the flow. See fig. S10 for constraints on how grain size varies with height above the bed, as well as through time.

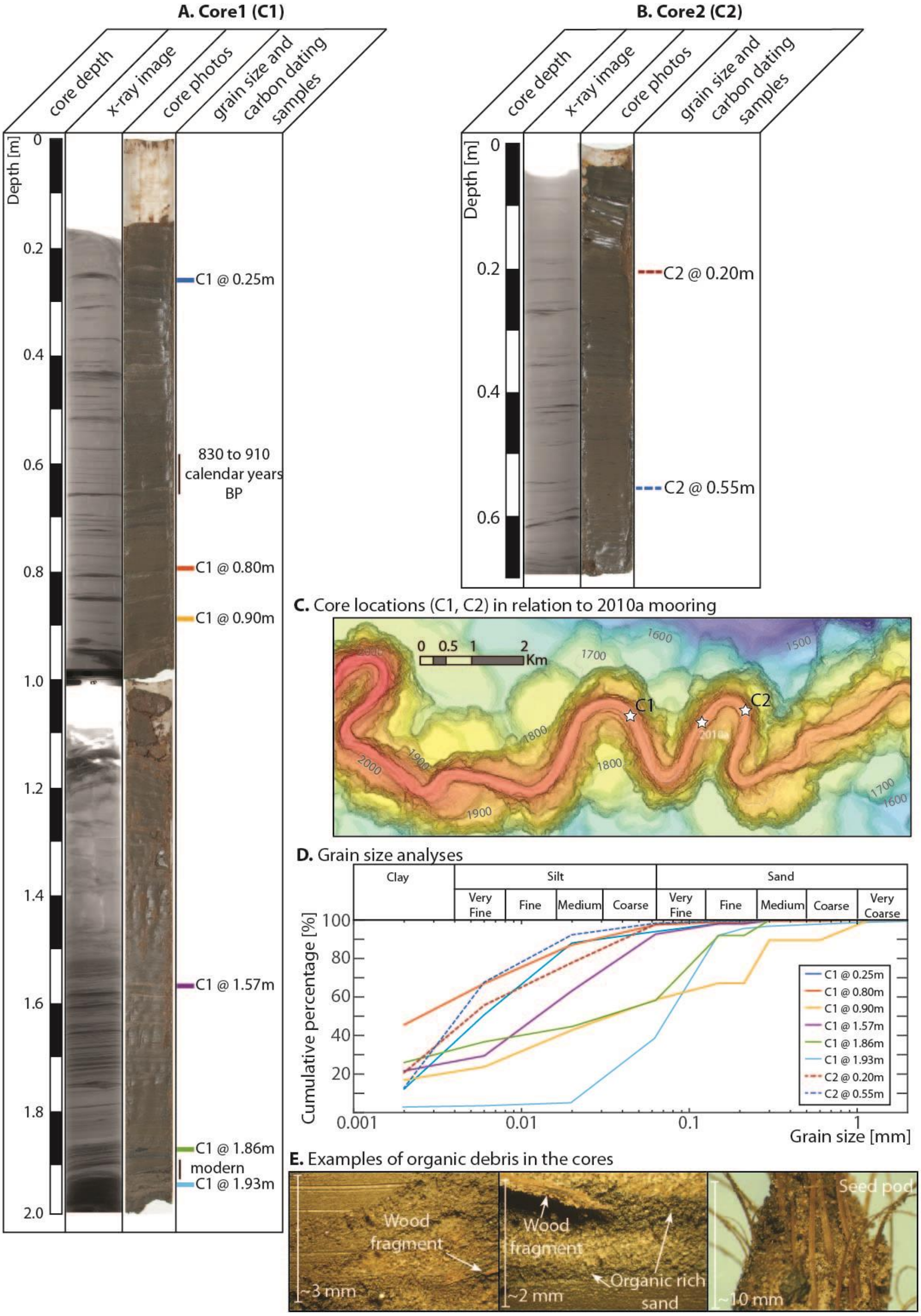

fig. S8. Cores from floor of Congo Canyon. (**A** and **B**) Core photo, x-ray images and sample locations. Dating in calendar years before present. (**C**) Core locations in relation to mooring site. (**D**) Grain size analyses. (**E**) Examples of organic debris from the cores.

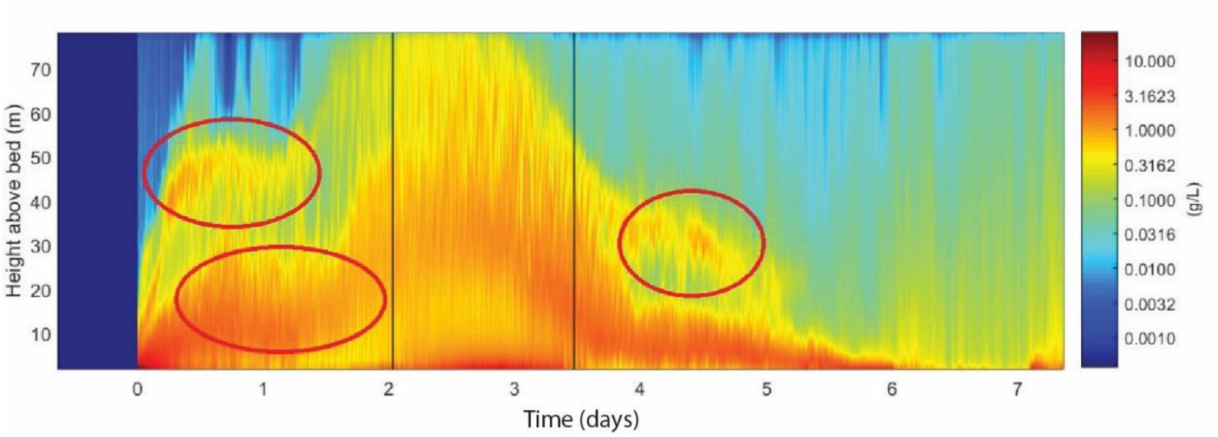

fig. S9. Sediment concentration (g/liter) derived using the ADCP backscatter magnitudes.

This analysis assumes that the flow contains a uniform grain size diameter of 4.23 µm. Potential artefacts are shown by red circles, where higher sediment concentrations overly lower sediment concentrations. These artefacts may result from variations in grain size within the flow, or from other factors, such as flow interactions with tidal currents.

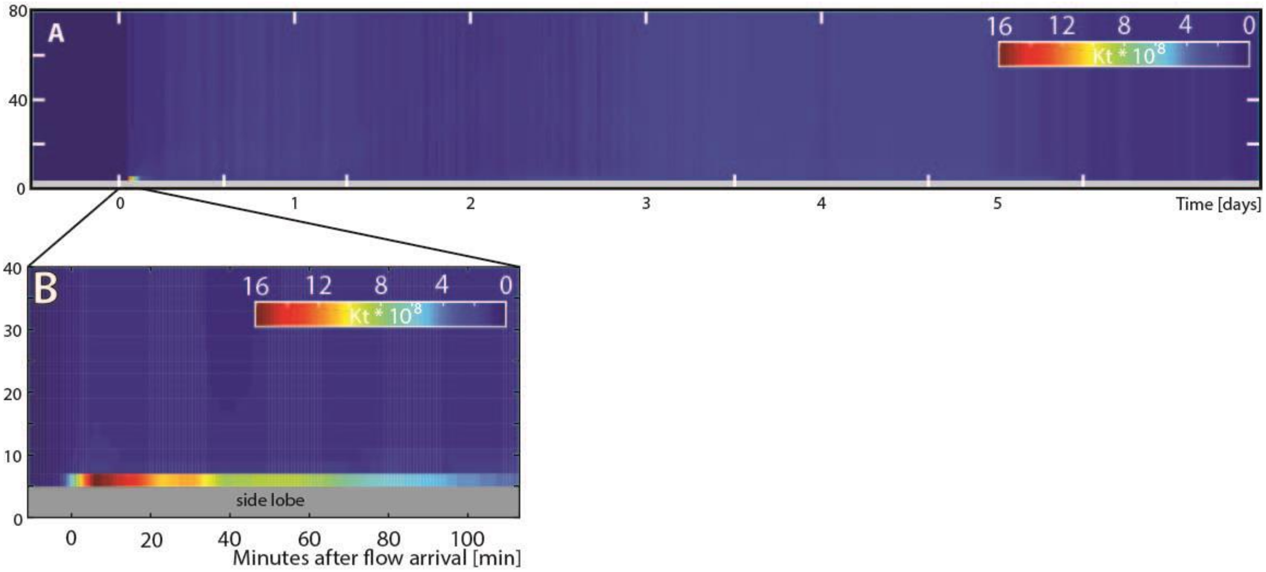

fig. S10. The calibration constant K_t. This constant is a measure of differences between the local grain size in the flow, and an assumed uniform grain size throughout the entire flow. This figure therefore shows that the lower 2-3 metres of the flow, during the initial 20-30 minutes of the event, is much coarser grained than the rest of the turbidity current. (**A**) Variation in K_t profiles throughout the whole 6 day long turbidity current. (**B**) Changes in K_t during the initial 2 hours of the flow, within the lower 40 metres.

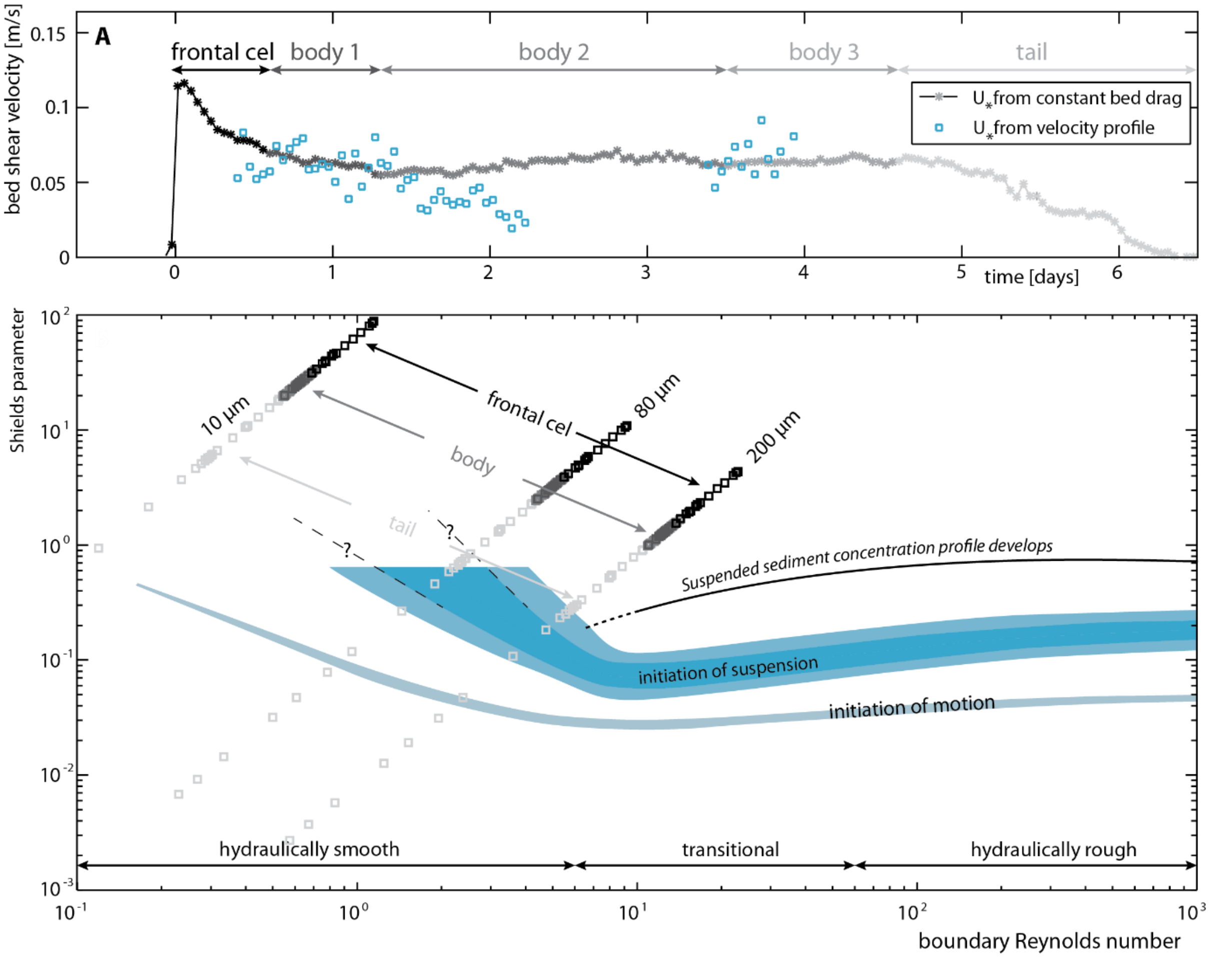

fig. S11. Bed shear stresses generated by the flow. (**A**) Bed shear velocities plotted over time. The blue square are shear velocities extracted from the gradient of the near-bed velocity profiles. The black and grey stars are shear velocities extracted from the velocity maximum based on a fixed bed drag coefficient. (**B**) Shield's diagram (modified from *28*) show which grain size can be transported either along the bed or in suspension. Here we plotted three grain sizes that are expected in the Congo Canyon (200 µm, 80 µm, and 10 µm). The plot shows that the frontal cell of the flow is the most capable of suspending sediment, and will erode sediment from the canyon floor to maintain a suspended sediment concentration profile. The values measured in the body of the flow are lower, but still sufficient to keep a suspended sediment concentration profile. At the tail of the flow the values drop below the initiation of suspension line, and will no longer be able to suspend the sediment in the flow. There are as yet few constrains on the initiation of suspension for clay-size particle, as indicated by the questions marks.

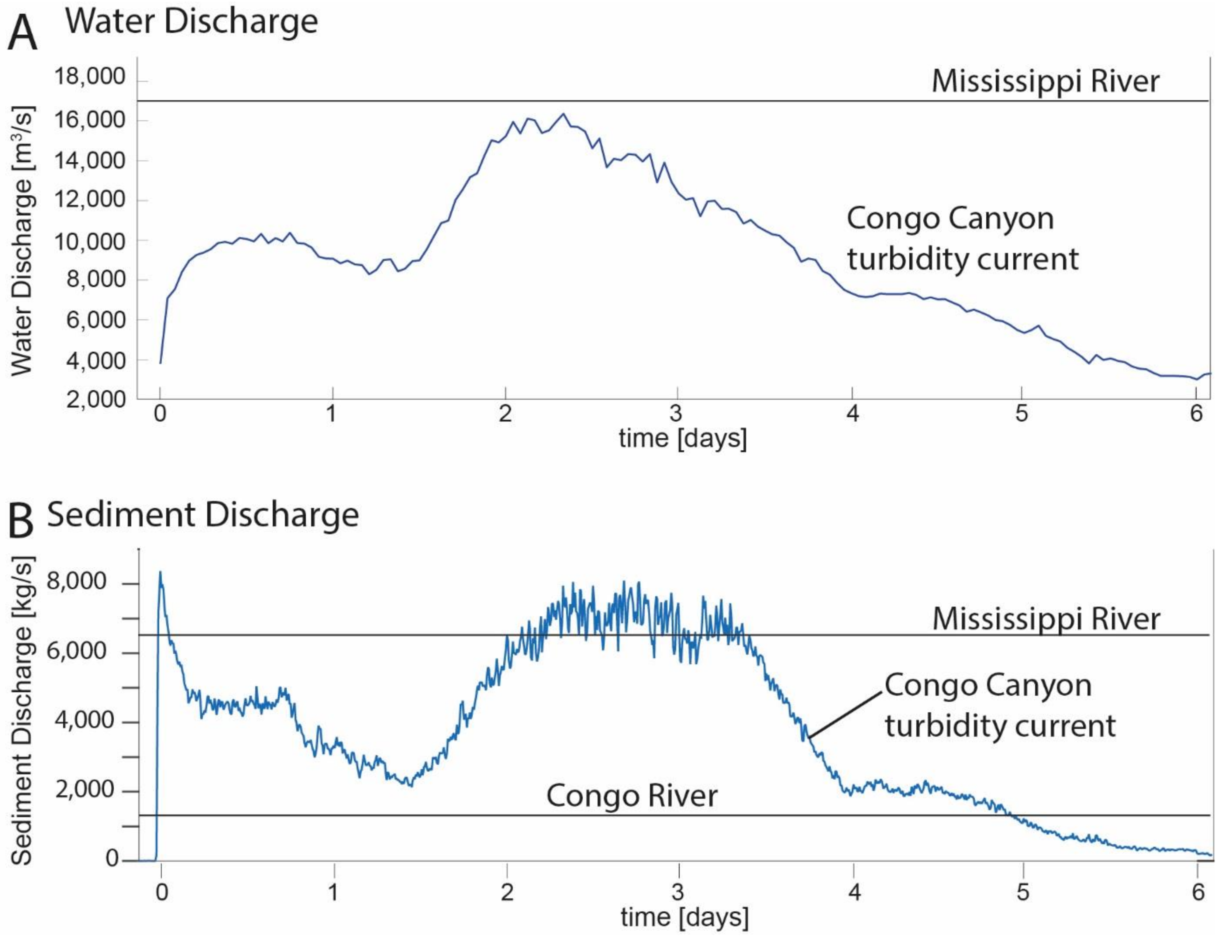

fig. S12. Comparisons of the instantaneous sediment and water discharges in the Congo Canyon turbidity current shown in Fig. 2, with the mean annual discharges of water and sediment in major rivers. (**A**) Water discharge in the Congo Canyon turbidity current (blue line) and Mississippi River (*4*) (black line). The Congo River has a mean annual discharge of 41,000 m^3/s *(4)*. (**B**) Sediment discharge within the turbidity current studied here, compared to the average annual sediment fluxes from the Mississippi and Congo Rivers (*4*).

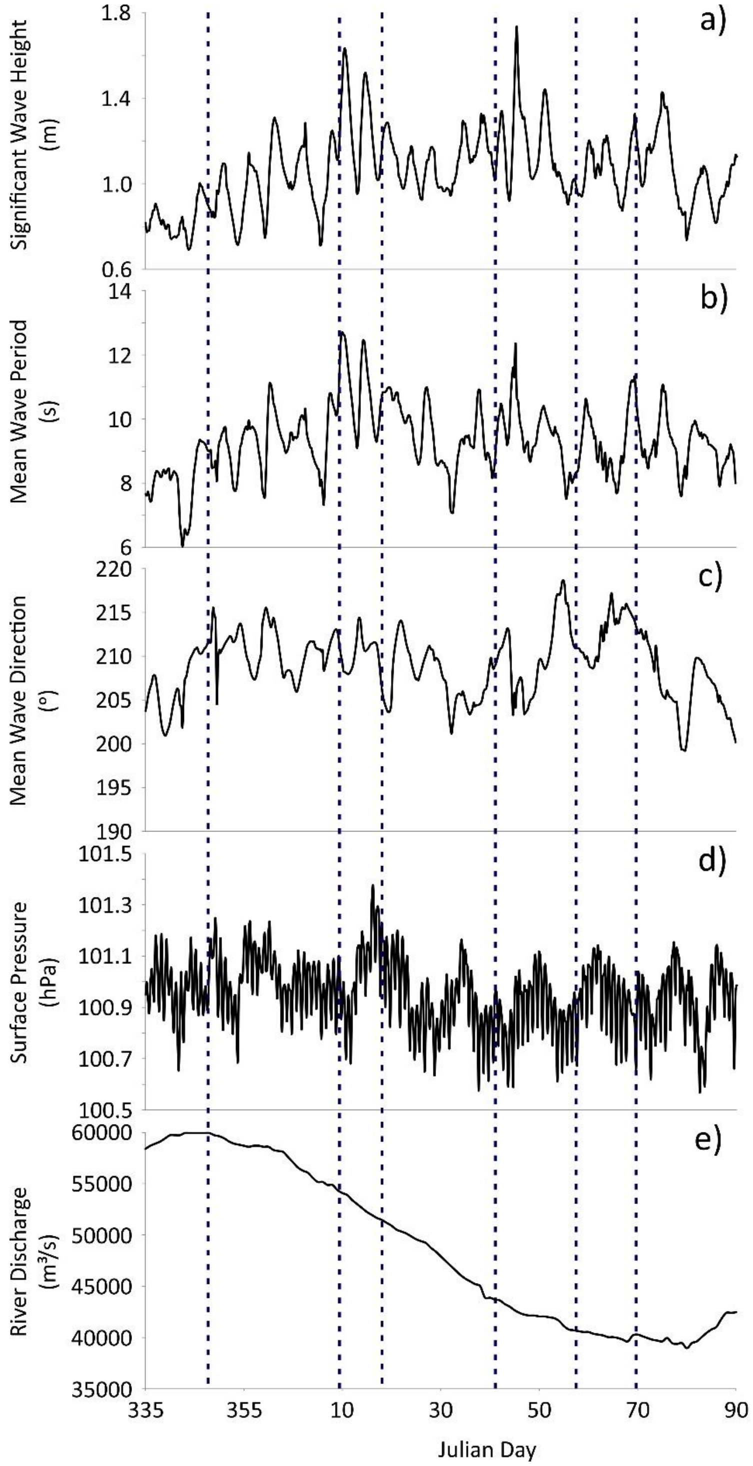

fig. S13. Comparison of turbidity current arrival times with possible triggering factors in the Congo Canyon. Turbidity current arrival times indicated by dashed lines. (**A**) Significant wave height. (**B**) Mean wave period. (**C**) Mean wave direction. (**D**) Surface pressure. (**E**) River discharge.

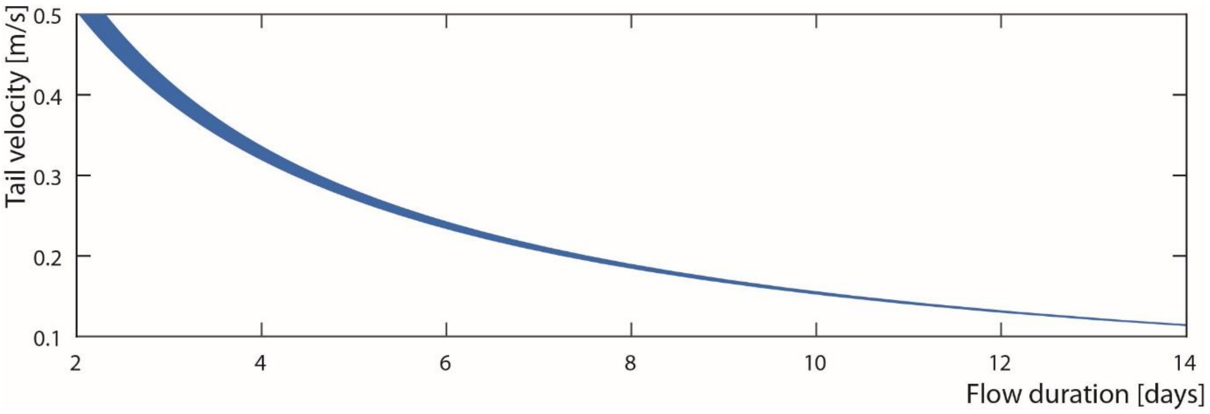

fig. S14. Increase in flow duration caused by flow stretching, which is due to a difference in the speed of the front and tail of the flow. The plot illustrates how variations in tail speed will affect the calculated degree of flow stretching. A runout distance of 170 km is assumed, together with frontal speeds of 1.2 m/s to 1.5 m/s. The width of blue line reflects that range of front speeds.

table S1. Flow durations, thicknesses, and peak velocity measured at heights in excess of 18 m above the bed in 2013.

Event No.	2013a* (up-canyon)			2013b Site (down-canyon)			Difference in flow duration (i.e. stretching) (days)
	Duration (days)	Elevation (m)	Peak speed at 18 m (m/s)	Duration (days)	Elevation (m)	Peak speed at 18 m (m/s)	
1	2.08	42.1	0.74	3.47	62.1	0.95	1.39
2	1.60	24.1	0.50	2.78	42.1	0.73	1.18
3	3.40	28.1	0.78	5.15	62.1	0.81	1.75
4	2.64	30.1	0.84	2.88	60.1	0.79	0.15
5	1.04	14.1	0.33	0.92	32.1	0.54	0.12
6	1.25	16.1	0.44	1.72	34.1	0.64	0.47
7	1.60	22.1	0.69	2.61	56.1	0.86	1.01
8	0.83	14.1	0.40	1.08	36.1	0.63	0.25
9	1.39	14.1	0.33	1.69	38.1	0.61	0.30
Mean ± standard deviation	1.76 ± 0.82	22.8 ± 9.54	0.56 ± 0.20	2.48 ± 1.32	47.0 ± 12.8	0.73 ± 0.13	0.74 ± 0.61

* The 2013a site is located 22 km up-canyon from the 2013b site.
